# Reversible formation of von-Willebrand-factor–platelet aggregates in microvascular blood flow

**DOI:** 10.1093/pnasnexus/pgaf375

**Published:** 2025-11-29

**Authors:** Alper Topuz, Masoud Hoore, Gerhard Gompper, Dmitry A Fedosov

**Affiliations:** Theoretical Physics of Living Matter, Institute for Advanced Simulation, Forschungszentrum Jülich, 52425 Jülich, Germany; Theoretical Physics of Living Matter, Institute for Advanced Simulation, Forschungszentrum Jülich, 52425 Jülich, Germany; Theoretical Physics of Living Matter, Institute for Advanced Simulation, Forschungszentrum Jülich, 52425 Jülich, Germany; Theoretical Physics of Living Matter, Institute for Advanced Simulation, Forschungszentrum Jülich, 52425 Jülich, Germany

**Keywords:** hemostasis, margination, aggregation, catch and slip bonds, mesoscopic modeling

## Abstract

Von Willebrand factor (VWF) and blood platelets play a key role in blood clotting by forming VWF–platelet aggregates. Using mesoscale hydrodynamic simulations, we study the behavior of these aggregates in microvascular blood flow. In microvessels, free VWF molecules and platelets migrate (or marginate) toward the vessel wall, due to the interaction with red blood cells under flow, such that their local concentration near the wall is substantially increased. Then, large shear rates near the wall facilitate the formation and growth of VWF–platelet aggregates. After reaching a certain size, the aggregates experience a hydrodynamic lift force and move away from the wall into the bulk flow, where local shear rates are relatively small, and dissociate reversibly into single components. Consequently, the described process repeats. In addition to the demonstration of recurrent formation and dissociation of VWF–platelet aggregates, our simulations show that shear activation of VWF and the interactions between VWF and platelets are the main determinants of this process. A significant change in the VWF shear activation or VWF–platelet interactions may readily lead to no aggregate formation or to the formation of irreversible aggregates.

Significance Statement Von Willebrand factor (VWF) is an essential protein that enables blood clotting and bleeding cessation at high shear rates prevalent in the microvasculature. VWF is a shear-sensitive protein such that its adhesive properties are activated only at high enough shear rates. Using hydrodynamic simulations, we show that VWF–platelet aggregates can spontaneously form and subsequently dissolve in the microvasculature under normal conditions, implying the existence of reversible VWF–platelet aggregates. However, under pathological conditions, VWF–platelet aggregation may become insufficient or irreversible. This indicates that blood clotting is a finely tuned process that properly functions under a wide range of blood flow conditions in the microvasculature.

## Introduction

The process of hemostasis or blood clotting is essential to stop bleeding in case of a vessel injury in order to maintain vascular integrity (1, 2). Even though blood platelets are one of the major players in the hemostatic process, their adhesion to an injury site becomes unreliable at high shear rates (larger than about $600\text{--}900\;s^{-1}$) (3, 4), which are often encountered within the microvasculature (5, 6). Under such high-shear-rate conditions, a long multimeric protein von Willebrand factor (VWF) plays a vital role, facilitating platelet capture and activation at the injury site (7–9). Malfunctions of VWF proteins often lead to either prolonged bleeding or undesired thrombotic events, some of which are described by different types of von Willebrand disease (10, 11).

VWF proteins are long concatemers of dimeric units, with a total length reaching tens of micrometers (12–14 ). Each VWF dimer contains an adhesive domain that is able to bind platelet receptors, or collagen exposed at the site of endothelial injury. Despite this capability for adhesion, VWF retains a globular form under static conditions due to self-attractive interactions, such that its adhesive sites are shielded and remain inactive (15, 16). In fact, VWF is a shear sensitive molecule, because it stretches only at high enough shear rates ($≳2,000\;s^{-}1$), which has been shown in microfluidic experiments (17) and by numerical simulations (18–20). More importantly, VWF stretching is directly associated with the activation of VWF for adhesion, as the adhesive domains become accessible in the stretched state (14, 17, 21, 22). The strong correlation between VWF stretching and its activation for adhesion has been demonstrated directly for a single VWF attached to a surface (21) and indirectly through the formation of surface-bound VWF–platelet aggregates (17, 22, 23). In the latter case, VWF–platelet aggregates at a surface form only at high enough shear rates, which are sufficient to stretch VWF molecules.

The formation of VWF–platelet aggregates at a surface has been studied in in vitro experiments (17, 22, 23) to better understand the role of VWF in primary hemostasis. Despite the fact that VWF-platelet aggregates form primarily at high shear rates, their aggregation is reversible upon flow cessation or substantial reduction (23–25). A simulation of VWFs with colloids mimicking platelets in shear flow, but in absence of red blood cells (RBCs) (23), has also confirmed shear-induced aggregation, which is reversible under flow cessation. The aggregation process critically depends on the strength of binding interactions between activated VWF molecules and colloids. In fact, atomic force microscopy experiments on the interaction of a single VWF adhesive domain with platelet receptors (26) have shown that such a bond has a nonmonotonic lifetime as a function of the applied force. After an initial decrease of the bond lifetime with increasing force, the lifetime jumps up by nearly two orders of magnitude, making the bond long-lived at higher applied forces. This behavior is characteristic for a so-called catch bond (i.e. bond lifetime increases with increasing applied force), which has also been observed in other interactions in biological systems (27, 28). Interactions between VWF and platelet can be affected by mutations (29, 30), making it interesting to investigate distinct interaction scenarios for the formation of VWF–platelet aggregates.

Even though there are multiple sources of VWF during hemostasis, blood plasma contains a certain amount of soluble VWFs (13, 31). This amount is generally sufficient to initiate wall-bound VWF networks or VWF–platelet aggregates, if VWF can adhere to the surface (23, 24, 32). This raises an interesting question whether dynamic VWF–platelet aggregates can spontaneously form in the microvasculature without any vessel damage (i.e. without a substrate for aggregate formation), since flow stresses in the microcirculation (at least in its arteriolar part) should be sufficient for VWF stretching with a consequent activation for adhesion. A further important aspect is whether such VWF–platelet aggregates would be long-lived or reversible.

We employ mesoscopic hydrodynamic simulations to demonstrate a surprising behavior of VWF–platelet aggregates in microvascular blood flow. The blood model rests on a particle-based hydrodynamics method, smoothed dissipative particle dynamics (SDPD) (33, 34), and mechanistic models of red blood cells (RBCs), platelets and VWFs. At the initial stage of blood flow in a microchannel, platelets and VWF chains migrate (or marginate) into a near-wall layer free of RBCs (called RBC free layer, or RBC-FL), such that their concentration is substantially enhanced near the wall (35). The margination phenomenon in microvessels has also been observed for leukocytes and drug delivery particles (35–38). Since VWFs are primarily stretched near the walls due to the large local shear rates, they bind flowing platelets, leading to a growth of VWF–platelet aggregates. When such aggregates become large enough, they migrate away from the walls into the bulk of blood flow due to hydrodynamic lift forces (39–41), where the flow stresses are significantly smaller than those near the walls. As a result, VWF-platelet aggregates dissociate near the channel center into single platelets and deactivated VWFs. Then, this process repeats itself, following the described steps, which include margination of platelets and VWFs, VWF stretching near the walls, the formation of VWF–platelet aggregates, with a consecutive migration away from the wall and dissociation. Thus, our simulations predict dynamic formation and dissociation of reversible VWF–platelet aggregates in microvascular flow. Furthermore, changes in VWF–platelet interactions, such as their catch- or slip-bond natures, can lead to the formation of irreversible aggregates, which possibly represent different types of pathological conditions related to VWF dysfunction or mutation.

## Models and methods

**Fig. 1.**
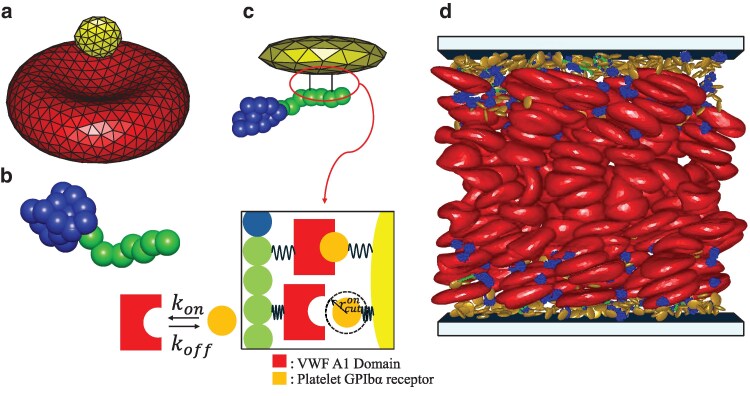
Illustration of the employed models and simulation setup. a) Membrane model of a RBC (red) and a platelet (yellow), whose surfaces are represented by a triangulated bead-spring network. The model incorporates shear and bending elasticity, and the conservation of surface area and cell volume. b) Polymer model of VWF whose globular part (blue) remains inactive (i.e. nonadhesive), while the stretched portion (green) is activated for adhesion. c) Adhesive bonds between activated monomers of VWF and platelet vertices, whose dynamics follows the prescribed on- and off-rates. d) The simulation domain of size 80μm×40μm×30μm with 360 RBCs, 768 platelets, and 384 VWFs. The snapshot shows that the majority of platelets and VWFs marginate into a near wall layer, where some VWFs are stretched by fluid stresses and become activated (marked green), enabling their adhesion to platelets and leading to the formation of aggregates.

Simulations of the dynamic formation of VWF–platelet aggregates in blood flow require models of blood cells (i.e. RBCs, platelets) and VWF molecules as well as a model to describe the dynamics of the suspending fluid (blood plasma). Figure 1 illustrates these models and shows a snapshot of the simulation domain, whose detailed description is provided below.

### Modeling fluid flow

Fluid flow is modeled by the smoothed dissipative particle dynamics (SDPD) method (33, 34), a mesoscopic hydrodynamics technique. SDPD is derived through a Lagrangian discretization of the Navier–Stokes equation with a consistent representation of thermal fluctuations (42). The SDPD fluid is modeled by a collection of particles, which correspond to small fluid volumes and interact through pairwise conservative, dissipative, and random forces. The conservative force imposes the equation of state $p=p_{0}(\rho /\rho_{0}{)}^{\gamma }-b$, and thus controls fluid compressibility (43). Here, *ρ* is the particle density, $\rho_{0}$ is a reference density, and $p_{0}$, *γ*, and *b* are selected parameters, which define the speed of sound $c^{2}=p_{0}\gamma /\rho_{0}$, and the basic level of fluid pressure $p_{0}-b$. The dissipative force controls fluid viscosity *η*, while the combination of dissipative and random forces forms a thermostat which imposes isothermal fluid conditions. All forces act within a cutoff radius $r_{c}$, and vanish beyond. We employ a version of SDPD which conserves angular momentum (34) in addition to the conservation of mass and linear momentum.

No-slip boundary conditions (BCs) at the channel walls are implemented by freezing a number of wall particles within a layer of thickness $r_{c}$, whose structural properties (e.g. radial distribution function) are the same as those of SDPD fluid. The frozen wall particles supply the appropriate SDPD interactions for near-wall fluid particles. To prevent penetration of the walls by fluid particles, bounce-back reflections at the fluid–solid interface are implemented. Furthermore, an adaptive shear force is applied to SDPD fluid particles within a near-wall layer of thickness $r_{c}$ (44, 45), since the dissipative interaction between fluid and wall particles may not be sufficient to strictly impose no-slip BCs. Blood cells and VWF molecules are coupled to the SDPD fluid through the dissipative interactions (46), which is similar to viscous coupling in the immersed boundary method (47). This implies that the viscosities of cell cytosol and blood plasma are identical.

### RBC and platelet models

Both RBCs and platelets are modeled by a triangulated network of $N_{v}$ particles (with coordinates $\{x_{i}{\}}_{i=1\cdots N_{v}}$) interconnected by bonds, which represents the cell surface (46, 48, 49), see Fig. 1(a). The potential energy of the network is defined as


(1) $$U(\{x_{i}\})=U_{s}+U_{b}+U_{a}+U_{v},$$

where $U_{s}$ is the bond potential, $U_{b}$ is the bending energy, and $U_{a}$ and $U_{v}$ correspond to area and volume conservation constraints, respectively. $U_{s}$ represents the membrane stretching elasticity. The curvature energy $U_{b}$ supplies bending resistance of the membrane, while the area $U_{a}$ and volume $U_{v}$ conservation constraints mimic the area incompressibility of the membrane and incompressibility of a cytosol, respectively. For further details, we refer the reader to Refs. (46, 48).

Elastic properties of both RBCs and platelets are characterized by the membrane shear modulus *μ* and the bending rigidity *κ*. Table 1 presents RBC and platelet characteristics in both simulation and physical units. The spontaneous curvature for RBCs is set to zero. Platelets assume an oblate shape with a diameter of 2μm and an aspect ratio of 0.3. Platelets are set to be about ten times stiffer (both larger *μ* and *κ*) than RBCs, in order to make platelets nearly undeformable. To keep a characteristic oblate shape, local spontaneous curvature at the surface of platelets has been set to the curvature of the original oblate shape.

**Table 1.** Parameters of RBCs and platelets in units of the effective RBC diameter $D_{r}=\sqrt{A_{r}/\pi }$ and the thermal energy $k_{B}T$ (T=310  K), and the corresponding values in physical units.

	RBC		Platelet	
Parameters	Scaled units	Physical units	Scaled units	Physical units
$N_{v}$	500		60	
*A*		$132.83\times 10^{-12}\;m^{2}$	$0.162D_{r}^{2}$	$6.85\times 10^{-12}\;m^{2}$
*V*	0.336 $D_{r}^{3}$	$92.4\times 10^{-18}\;m^{3}$	0.0041 $D_{r}^{3}$	$1.127\times 10^{-18}\;m^{3}$
*μ*	$4.77\times 10^{4}$ $k_{B}T/D_{r}^{2}$	$4.83\times 10^{-6}\;N/m$	$4.77\times 10^{5}$ $k_{B}T/D_{r}^{2}$	$4.83\times 10^{-5}\;N/m$
*κ*	70 $k_{B}T$	$3\times 10^{-19}\;J$	700 $k_{B}T$	$3\times 10^{-18}\;J$
$k_{d}$	$4.23\times 10^{4}$ $k_{B}T/D_{r}^{2}$	$4.28\times 10^{-6}\;N/m$	$4.23\times 10^{5}$ $k_{B}T/D_{r}^{2}$	$4.28\times 10^{-5}\;N/m$
$k_{a}$	$2.07\times 10^{6}$ $k_{B}T/D_{r}^{2}$	$2.1\times 10^{-4}\;N/m$	$3.81\times 10^{6}$ $k_{B}T/D_{r}^{2}$	$3.86\times 10^{-4}\;N/m$
$k_{v}$	$1.37\times 10^{7}$ $k_{B}T/D_{r}^{3}$	$213.28\;{N/m}^{2}$	$2.75\times 10^{7}$ $k_{B}T/D_{r}^{3}$	$428.11\;{N/m}^{2}$

$N_{v}$ is the number of membrane vertices, *A* is the membrane area, *V* is the volume, *μ* is the membrane shear modulus, *κ* is the membrane bending rigidity, and $k_{d}$, $k_{a}$, and $k_{v}$ are the local area, global area, and volume constraint coefficients, respectively. In all simulations, we have chosen $A_{r}=132.83$ and $k_{B}T=0.1$, which implies that $D_{r}=6.5$.

### Shear activated VWF

VWF is a self-attractive shear-activated protein, such that it remains nonadhesive in a globular form at low fluid stresses or in the absence of flow, but it becomes adhesive upon stretching at high enough shear stresses (17, 21, 31). In our simulations, VWF is represented by a self-avoiding bead-spring model of $N_{m}$ monomers ($N_{m}=30$) [see Fig. 1(b)] with attractive interactions between monomers (18, 19, 50) connected by finite-extensible nonlinear elastic (FENE) springs to form a linear polymer chain, with bond potential


(2) $$U_{\mathrm{FENE}}(r)=-\frac{k_{s}}{2}r_{\max}^{2}\ln\;\left(1-{\left(\frac{r}{r_{\max}}\right)}^{2}\right),$$

where $k_{s}$ is the spring stiffness, $r_{\max}=2\sigma$ is the maximum spring extension ($\sigma =0.077D_{r}$ is the bead diameter), and *r* is the distance between two consecutive monomers. $k_{s}$ is set to $25,000k_{B}T/\sigma^{2}$ in all simulations, making the connecting springs nearly inextensible. Self-avoidance and self-attraction within the polymer are implemented by the 12-6 Lennard–Jones (LJ) potential


(3) $$U_{\mathrm{LJ}}(r)=4\epsilon \left[{\left(\frac{\sigma }{r}\right)}^{12}-{\left(\frac{\sigma }{r}\right)}^{6}\right],$$

where $\epsilon =16k_{B}T$ controls the attraction strength. The LJ interaction for monomers within the same VWF is cut beyond $r_{LJ}=2.5\sigma$. VWF chains are coupled to the SDPD fluid by a frictional force (51, 52).

This VWF model keeps a globular configuration at low shear rates and stretches out at large enough shear rates ($≳2,000\;s^{-}1$) (18–20, 52), similarly to the observed behavior of VWF in shear flow (17, 21, 31). In the stretched state, VWF becomes adhesive, such that it can bind to platelet receptors. The activation of adhesion depends on the local stretching of VWF model in flow (52), which is described by two geometrical criteria. The first criterion concerns the local angle $\theta_{i-1,i,i+1}$ between two consecutive bonds linking neighboring monomers i−1, *i*, and i+1, and is expressed as $\theta_{i-1,i,i+1}\geq \theta_{\mathrm{thres}}$ for $2<i<N_{m}-1$, with a threshold angle $\theta_{\mathrm{thres}}$ that defines the degree of local stretching of VWF. This condition is always assumed to be satisfied for the first and the last bead in the VWF. The second condition monitors the presence of nonbonded neighboring monomers $N_{\mathrm{neigh}}$ within the polymer as $N_{\mathrm{neigh}}=0$ for $r_{ij}\leq R_{\mathrm{thres}},\;j\neq i,i\pm 1$, prohibiting the activation of monomers within a globule configuration. Here, $R_{\mathrm{thres}}$ is a threshold radius. In all simulations, $\theta_{\mathrm{thres}}=150^{o}$ and $R_{\mathrm{thres}}=1.2\sigma$ are employed. The sensitivity of VWF activation to the choice of $\theta_{\mathrm{thres}}$ and $R_{\mathrm{thres}}$ has been investigated in Ref. (20). An inactive monomer is activated whenever both conditions are satisfied. Consistently, any activated VWF monomer is deactivated whenever one or the both criteria are no longer met.

### Adhesion interactions

For molecular interactions, the probability of bond rupture usually increases with increasing applied forces. This type of behavior is well described by a slip-bond model, which assumes a positive correlation between the rupture probability and the applied force. A high rupture probability implies a short lifetime of the bond, such that the lifetime of a slip bond decreases with increasing applied force. However, experiments (53–55) have demonstrated that the lifetime of certain ligand–receptor interactions may increase as the applied force is elevated, at least for some intermediate range of force magnitudes. In this case, the bond is referred to as a catch bond (53). Clearly, any physical bond will eventually rupture at large enough forces. Therefore, the catch behavior of a real bond should rather be considered as a dual catch-slip behavior, where the lifetime first increases (i.e. catch behavior) and then decreases (i.e. slip behavior) with increasing applied force. Even though the catch-slip behavior has been found for only a few ligand–receptor pairs (54, 55), the existence of this type of bonds is well accepted in the context of biological systems (55). Importantly, adhesion of VWF to platelet GPIb*α* receptor exhibits the catch-slip behavior (26, 29, 56).

Adhesive interactions between activated VWF monomers and platelet vertices are implemented through the formation of bonds with predefined association, $k_{\mathrm{on}}$, and dissociation, $k_{\mathrm{off}}$, rates [see Fig. 1(c)]. Formed bonds are modeled by the harmonic potential $U_{b}=k_{b}(r-r_{0}{)}^{2}$, where $k_{b}$ is the bond strength and $r_{0}$ is its equilibrium length. Probabilities for bond formation $P_{\mathrm{on}}$ and dissociation $P_{\mathrm{off}}$ are determined by the rate equations (57)


(4) $$\frac{dP_{\mathrm{on}}}{dt}=-k_{\mathrm{on}}P_{\mathrm{on}},\;\text{for }r\leq r_{\mathrm{cut}}^{\mathrm{on}},$$

(5) $$\frac{dP_{\mathrm{off}}}{dt}=-k_{\mathrm{off}}P_{\mathrm{off}},\;\text{for }r\leq r_{\mathrm{cut}}^{\mathrm{off}},$$

where $r_{\mathrm{cut}}^{\mathrm{on}}$ and $r_{\mathrm{cut}}^{\mathrm{off}}$ are the cutoff ranges for bond association and dissociation, respectively. Note that $P_{\mathrm{on}}=0$ for $r>r_{\mathrm{cut}}^{\mathrm{on}}$ and $P_{\mathrm{off}}=1$ for $r>r_{\mathrm{cut}}^{\mathrm{off}}$. In simulations, we assume $k_{\mathrm{on}}$ to be constant, while $k_{\mathrm{off}}$ follows the aforementioned catch-slip behavior. This is described by the two-pathway model (58), which assumes two force-dependent barriers for bond dissociation,


(6) $$k_{\mathrm{off}}(r)=k_{c}^{0}\exp\;\left(\frac{\lambda_{c}(r-x_{\mathrm{eq}})\delta_{c}}{k_{B}T}\right)+k_{s}^{0}\exp\;\left(\frac{\lambda_{s}(r-x_{\mathrm{eq}})\delta_{s}}{k_{B}T}\right),$$

where the first term represents a catch-bond dissociation rate, while the second term corresponds to a slip-bond rate. Here, $k_{c}^{0}$ and $k_{s}^{0}$ are the catch and slip equilibrium off-rates. $\lambda_{c}$ and $\lambda_{s}$ represent the strengths of the catch and slip contributions, respectively, and $\delta_{c}=x_{c}-x_{\mathrm{eq}}$ and $\delta_{s}=x_{s}-x_{\mathrm{eq}}$, where $x_{c}$, $x_{s}$, and $x_{\mathrm{eq}}$ are the catch, slip, and equilibrium characteristic lengths, respectively. Note that the condition $x_{c}<x_{\mathrm{eq}}<x_{s}$ should be satisfied, so that the catch part dominates for $r<x_{\mathrm{eq}}$, while the slip part dominates for $r>x_{\mathrm{eq}}$.

### Simulation setup

Blood flow is simulated within a rectangular box of dimension $L_{x}\times L_{y}\times L_{z}=80\;\mu m\times 40\;\mu m\times 30\;\mu m$ with 360 RBCs, 768 platelets, and 384 VWFs. Periodic BCs are assumed in *x* and *z* directions, while wall boundaries with no-slip BCs are set in *y* direction. The volume fraction of RBCs (or hematocrit) is ∼42%, and platelets and VWFs account for about 1.7 and 0.8% by volume, respectively. Note that the simulated volume fractions of VWFs and platelets are higher than under physiological conditions, in order to allow the formation of large enough aggregates with a limited number of platelets and VWFs within the simulation domain. The suspending fluid is modeled by $4.8\times 10^{5}$ SDPD particles, corresponding to the number density of $n=16.875/r_{c}^{3}$ with $r_{c}=0.23D_{r}$. The SDPD fluid parameters are $p_{0}=b=33,750k_{B}T/r_{c}^{3}$, $\rho_{0}=mn$ with the particle mass *m* (m=1 in simulations), and γ=7. Fluid viscosity is set to η=100, which defines a time scale $\tau =\eta D_{r}/\mu_{r}$. For the blood plasma viscosity of $\eta =1.2\times 10^{-3}\;\mathrm{Pa}\cdot s$, this yields τ≈0.0016 s. The flow is driven by a pressure gradient of $\Delta P/L_{x}=fn=81k_{B}T/r_{c}^{4}$, corresponding to a force *f* applied to each SDPD fluid particle in the flow direction along the *x* axis.

Excluded-volume interactions between RBCs, platelets, and VWFs are implemented through the repulsive part of the LJ potential in Eq. (3). For all repulsive interactions, $\epsilon =16k_{B}T$ and $r_{LJ}=2^{1/6}\sigma$. However, $\sigma =0.077D_{r}$ for VWF–VWF interactions, $\sigma =0.062D_{r}$ for RBC–VWF and platelet–VWF repulsion, and $\sigma =0.046D_{r}$ for RBC–RBC, platelet–platelet, and RBC–platelet interactions, due to slight differences in the discretization resolution of various suspended components.

**Fig. 2.**
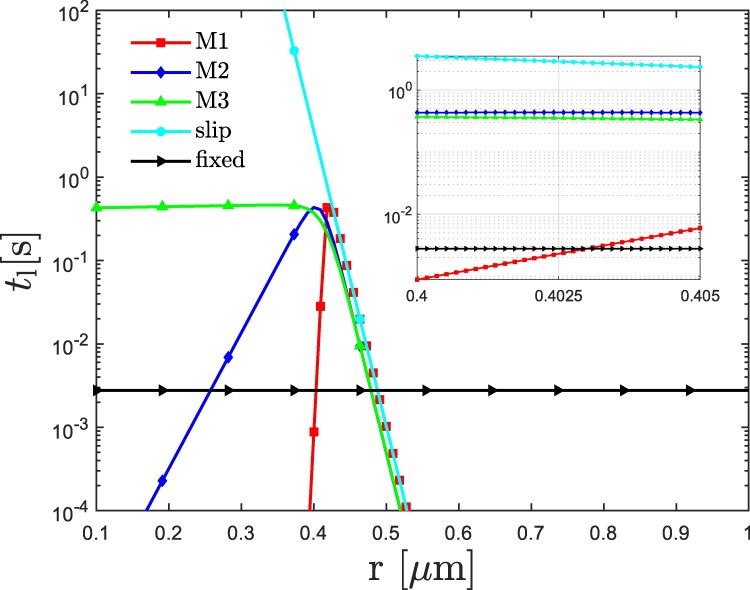
Comparison of lifetimes $t_{l}=1/k_{\mathrm{off}}$ for different bond types (see Table 2) as a function of bond length *r*. The inset shows the behavior of same curves near the average bond length.

In the model, platelet vertices represent receptors, and activated VWF monomers represent ligands. Each adhesive site on VWF or platelet can accommodate only a single bond. Figure 2 shows the lifetime $t_{l}=1/k_{\mathrm{off}}$ of different bond types as a function of the bond length *r*. The parameters of different bond types are given in Table 2. Note that these parameters represent effective bonds and do not correspond to molecular bond properties, because the minimally resolved length scale in our model is significantly above the molecular level. The bond parameters have been calibrated in separate shear flow simulations without RBCs, where the formation of stable VWF–platelet aggregates was targeted at high shear rates, with their consecutive dissociation at low shear rates. Furthermore, for all bond types, $k_{b}=8.45\times 10^{6}k_{B}T/D_{r}^{2}$, $r_{0}=0.062D_{r}$, $k_{\mathrm{on}}=5760/\tau$, $r_{\mathrm{cut}}^{\mathrm{on}}=0.068D_{r}$, and $r_{\mathrm{cut}}^{\mathrm{off}}=0.23D_{r}$. The bond parameters are calibrated for the catch-slip model M1 to properly reproduce reversible aggregation of VWFs and platelets. The other two catch-slip models (M2 and M3) represent intermediate lifetimes between the catch-slip model M1 and the slip bond model.

**Table 2.** VWF–platelet interaction parameters for different bond types.

Bond type	$k_{c}^{0}$	$x_{c}$	$x_{\mathrm{eq}}$	$k_{s}^{0}$	$x_{s}$
Catch-slip M1	$5.76\times 10^{-1}/\tau$	$0.0385D_{r}$	$0.062D_{r}$	$5.76\times 10^{-4}/\tau$	$0.067D_{r}$
Catch-slip M2	$1.73\times 10^{-2}/\tau$	$0.0517D_{r}$	$0.054D_{r}$	$1.73\times 10^{-5}/\tau$	$0.059D_{r}$
Catch-slip M3	$3.46\times 10^{-3}/\tau$	$0.05098D_{r}$	$0.051D_{r}$	$3.46\times 10^{-6}/\tau$	$0.056D_{r}$
Slip	0	$0.062D_{r}$	$0.062D_{r}$	$5.76\times 10^{-4}/\tau$	$0.067D_{r}$
Fixed	$5.77\times 10^{-1}/\tau$	$0.062D_{r}$	$0.062D_{r}$	0	$0.062D_{r}$

In all cases, $\lambda_{c}=\lambda_{s}=1.06\times 10^{5}k_{B}T/D_{r}^{2}$.

## Results

**Fig. 3.**
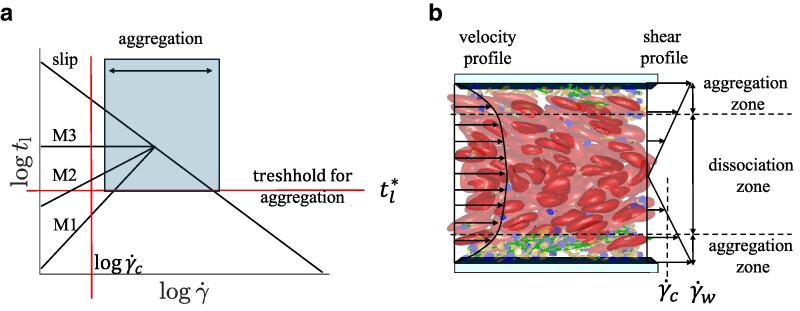
A schematic of the conditions for the formation of VWF–platelet aggregates. a) Qualitative dependence of VWF–platelet aggregate formation as a function of shear rate and the lifetime of bonds between VWFs and platelets. Lifetime for several bond scenarios, including catch and slip bonds, is shown. ${\dot{\gamma }}_{c}$ denotes a critical shear rate for VWF stretching and activation, while $t_{l}^{*}$ represents a critical lifetime of bonds that is long enough to maintain stable aggregates. Thus, VWF–platelet aggregates form under the conditions of $\dot{\gamma }≳{\dot{\gamma }}_{c}$ and $t_{l}≳t_{l}^{*}$. b) A snapshot of modeled blood flow with a potential aggregation zone in the vicinity of the walls. Near the center of the channel, no aggregates can form and existing VWF–platelet aggregates would dissociate, which is marked by the dissociation zone. The snapshot shows RBCs (red), platelets (yellow), and VWFs with inactive (blue) and active (green) monomers.

The formation of VWF–platelet aggregates in flow relies on two basic requirements. First, flow stresses need to be large enough to stretch VWF globules, and therefore, activate VWFs for adhesion. Second, bonds formed between activated VWFs and platelets have to be durable enough to maintain the temporary stability of the formed aggregates. This means that their lifetime has to be long enough to prevent a quick breakage of bonds within the aggregate. Figure 3(a) schematically illustrates bond lifetimes for different models as a function of shear rate $\dot{\gamma }$ (or stress), which is expected to determine local bond extension (compare Fig. 2). The shaded area in Fig. 3(a) denoted as “aggregation” corresponds to $\dot{\gamma }≳{\dot{\gamma }}_{c}$ and $t_{l}≳t_{l}^{*}$, where ${\dot{\gamma }}_{c}$ represents a critical shear rate for VWF stretching and activation, and $t_{l}^{*}$ denotes a critical lifetime of bonds that is long enough to ensure stable aggregates. Thus, in this area, both conditions are satisfied and the formation of stable VWF–platelet aggregates can take place.

The VWF model from Refs. (20, 52) shows a shear-dependent stretching of VWF at ${\dot{\gamma }}_{c}\approx 2,000\;s^{-1}$, in agreement with experimental observations (17, 21, 31). Furthermore, the parameters of the catch-slip model M1 in Table 2 are calibrated in simulations of pure shear flow using a suspension of only platelets and VWFs, such that stable VWF–platelet aggregates form for $\dot{\gamma }≳{\dot{\gamma }}_{c}$. Using the catch-slip model M1, we have also verified that VWF–platelet aggregates dissolve when the shear rate is reduced below ${\dot{\gamma }}_{c}$, which is consistent with the reversible aggregation of platelets and VWFs observed in experiments (21, 23, 56). The dissociation of aggregates at $\dot{\gamma }<{\dot{\gamma }}_{c}$ is primarily due to a significant reduction in bond lifetime in comparison to $\dot{\gamma }>{\dot{\gamma }}_{c}$, see the catch-slip model M1 in Fig. 3(a). In contrast, the slip-bond case in Fig. 3(a) results in the formation of irreversible VWF–platelet aggregates, since the dissociation of aggregates at $\dot{\gamma }<{\dot{\gamma }}_{c}$ is limited by long lifetimes. Theoretically, VWF–platelet aggregates based on the slip-bond model would also eventually dissolve at $\dot{\gamma }<{\dot{\gamma }}_{c}$, however, their disaggregation time is several orders of magnitude longer than that for the case of catch-slip M1 bonds. Thus, the dissociation of aggregates for the slip-bond case does not occur during typical simulation times, and this type of aggregates is considered to be irreversible.

Following the two conditions for the formation of VWF–platelet aggregates, blood flow in a channel (or a vessel) can be divided in two zones depending on the local shear rate, which is schematically shown in Fig. 3(b). The region near the walls with $\dot{\gamma }>{\dot{\gamma }}_{c}$ is called an aggregation zone, where the formation of aggregates is possible. The region around the center of the channel is referred to as a dissociation zone, where formed VWF–platelet aggregates are expected to break apart (at least for the case of catch-slip model M1). Note that the formation of aggregates is not possible in the dissociation zone due to small local shear stresses, which does not facilitate the activation of VWFs. Clearly, when blood flow in a vessel is weak enough, the aggregation zone may completely disappear due to small local shear rates near the walls, leading to no aggregate formation under such flow conditions.

### Reversible aggregate formation in blood flow

**Fig. 4.**
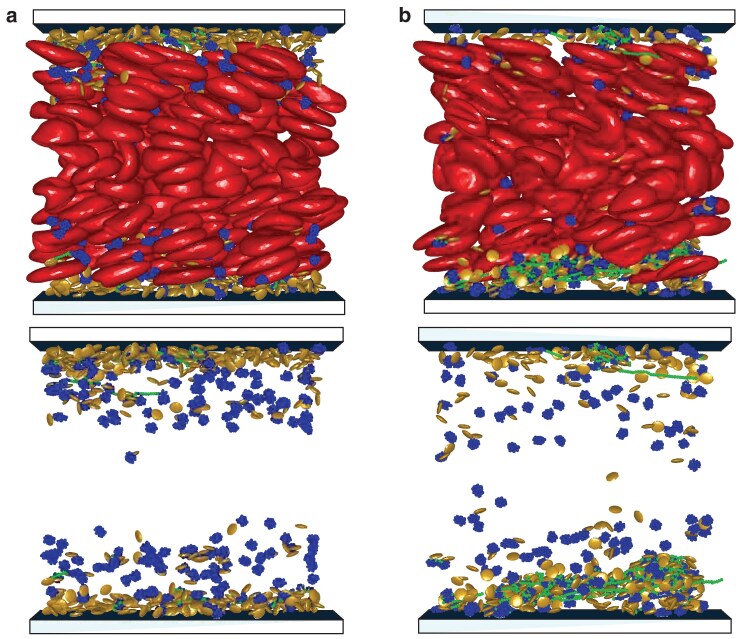
Migration and VWF-platelet aggregation in blood flow. a) Margination of platelets and VWFs into the RBC-FL, leading to their localization near the walls. b) Formation of VWF–platelet aggregates near the walls after the stretching and activation of marginated VWFs, see Movie S1. The snapshots show RBCs (red), platelets (yellow) and VWFs with inactive (globular, blue) and active (stretched, green) monomers. The bottom row shows the corresponding snapshots with only platelets and VWFs displayed.

We first focus on the case of normal functioning of VWF, which is mimicked by the catch-slip model M1. At the start of the simulation, all VWFs have a globular form (i.e. nonadhesive) and are uniformly distributed within the simulation domain together with platelets and RBCs, without any overlaps and any bonded structures. As the driving force is applied to initiate blood flow, RBCs migrate toward the channel center, while platelets and VWF globules slowly migrate (or marginate) toward the channel walls, see Fig. 4(a). As a result, the concentration of platelets and VWFs in the RBC-free layer (RBC-FL) increases many folds, providing favorable conditions for the formation of VWF–platelet aggregates.

In addition to the increased concentration of platelets and VWFs near the walls due to margination, the shear rate close to the walls is the largest in blood flow. In our simulations, the wall shear rate is ${\dot{\gamma }}_{w}=fnL_{y}/(2\eta )\approx 1,152\;s^{-1}$. Note that it is smaller than ${\dot{\gamma }}_{c}\approx 2,000\;s^{-1}$ predicted for VWF stretching in pure shear flow. In blood flow within the RBC-FL, VWF stretches and becomes activated at much lower shear rates, due to its quasiconfinement between the wall and flowing RBCs (35). As a result, marginated VWFs quickly stretch within the RBC-FL and start binding nearby platelets, resulting in the formation of VWF–platelet aggregates [see Fig. 4(b)]. These aggregates grow in size by binding other activated VWFs and platelets or through the coalescence of two or more smaller aggregates.

**Fig. 5.**
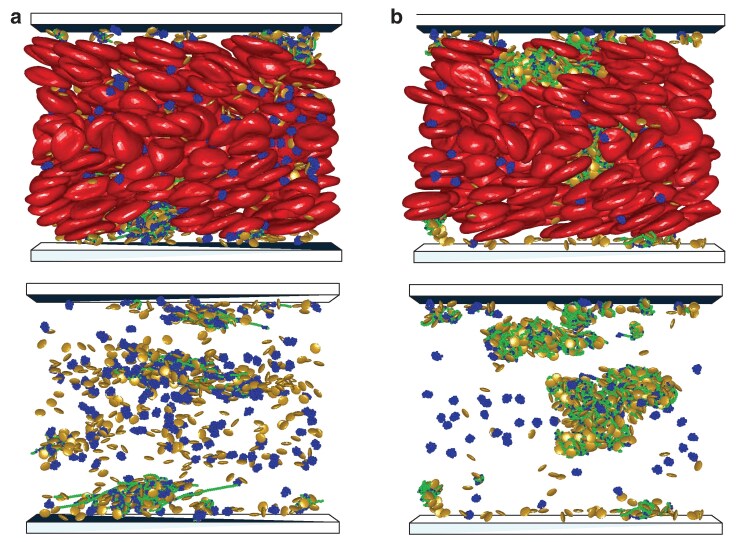
VWF–platelet aggregates in blood flow. a) The catch-slip model M1 leads to the dissociation of aggregates, as they migrate away from the walls and enter the dissociation zone with $\dot{\gamma }<{\dot{\gamma }}_{c}$. In this case, reversible aggregation of platelets and VWFs is observed. See also Movie S2. b) The slip bond model leads to the formation of irreversible VWF–platelet aggregates. They migrate toward the channel center and show no significant dissociation during the simulation time (see Movie S3). The bottom row shows the corresponding snapshots with only platelets and VWFs displayed.

As the aggregates grow in size within the RBC-FL, they acquire a significant lift force due to hydrodynamic interactions with the wall (39–41), and start migrating away from the wall. The migration away from the wall occurs roughly when the aggregate size exceeds the thickness of the RBC-FL. As VWF–platelet aggregates move toward the channel center, they experience a significant reduction in flow stresses, which results in their dissociation into separate unbound platelets and VWFs, as shown in Fig. 5(a). This behavior is consistent with the proposition of aggregation and dissociation zones illustrated in Fig. 3(b), depending on the lateral position of VWF–platelet aggregates in the channel. Interestingly, the described process repeats after the dissociation of aggregates. Freed platelets and VWFs marginate into the RBC-FL again, and participate in the formation of new aggregates near the walls. As a result, bond interactions described by the catch-slip model M1 lead to the formation of reversible aggregates with a continuous interchange between aggregation and dissociation, as a function of the lateral position within the channel.

### Irreversible aggregates in blood flow

As we mentioned before, the slip bond model is expected to result in irreversible aggregation. At the start of the simulation, the behavior of the slip bond model is similar to that with the catch-slip model M1, as described in the previous section “Reversible aggregate formation in blood flow”. Free platelets and VWFs first marginate into the RBC-FL, where VWFs stretch and become activated, followed by the formation of VWF–platelet aggregates. When the aggregates are large enough, they migrate toward and eventyually reach the channel center, where no significant dissociation is observed, as shown in Fig. 5(b). In fact, these aggregates eventually reach the channel center, where the shear stresses are quite small, but do not exhibit any dissociation for the rest of the simulation in Fig. 5(b). The formation of new aggregates near the walls also stops, because the numbers of platelets and VWFs are fixed in the simulation, and most of them are already part of the existing irreversible aggregates.

### Dynamic characteristics of different aggregate types

**Fig. 6.**
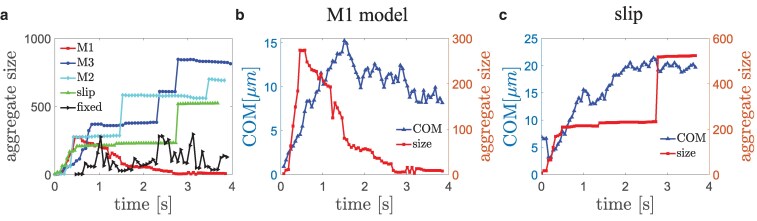
Size of aggregates and their position in the channel as a function of time. a) Aggregate size (or the total number of platelets and VWFs forming it) for different bond models, including catch-slip models M1 (squares, red), M2, (blue circles), and M3 (diamonds, cyan), the slip model (triangles, green), and the model with a fixed off rate (triangles, black). b) Aggregate size and the distance of its center-of-mass (COM) to the wall for catch-slip model M1. c) Aggregate size and the distance of its COM to the wall for the slip model.

To quantify the behavior of VWF–platelet aggregates for different bond models, we analyze the size of aggregates (i.e. the total number of VWFs and platelets), their lateral position within the channel and their asphericity, stretching of VWFs, and the average lifetime of bonds within the aggregates. Figure 6(a) presents aggregate sizes as a function of time for different bond models (see Fig. 2 and Table 2). Note that initial conditions are the same in all simulations, so that the processes of margination and aggregate formation near the wall at short times are similar for all cases [see Fig. 6(a)], except for the model with a fixed off-rate. It is apparent that the chosen fixed value of $k_{\mathrm{off}}$ is too large to sustain large aggregates consisting of more than about 200 platelets and VWFs. Another striking difference is that the aggregate size for the catch-slip model M1 starts to decay after the initial growth, while for the M2, M3, and slip models, the size monotonically increases. The onset of the decay for the M1 model coincides with aggregate migration away from the wall and entering the flow dissociation zone, as can be seen from the center-of-mass (COM) position of the aggregate with respect to the wall in Fig. 6(b). After the migration toward the channel center, the aggregate fully dissociates. The time of aggregate dissociation corresponds to several seconds, as can be seen from the decay of aggregate size. In contrast, the size of aggregates with slip bonds increases monotonically, even after the aggregate left the wall, see Fig. 6(c). This quantitatively confirms the irreversibility of aggregation with slip bonds, at least over the time scale of our simulations. The sudden jumps in aggregate size in Fig. 6(c) correspond to merging of two separate aggregates as they spontaneously encounter each other in flow. A very similar behavior is also observed for the catch-slip models M2 and M3 [see Fig. 6(a)], such that the formed aggregates are nearly irreversible on the time scale of simulations.

**Fig. 7.**
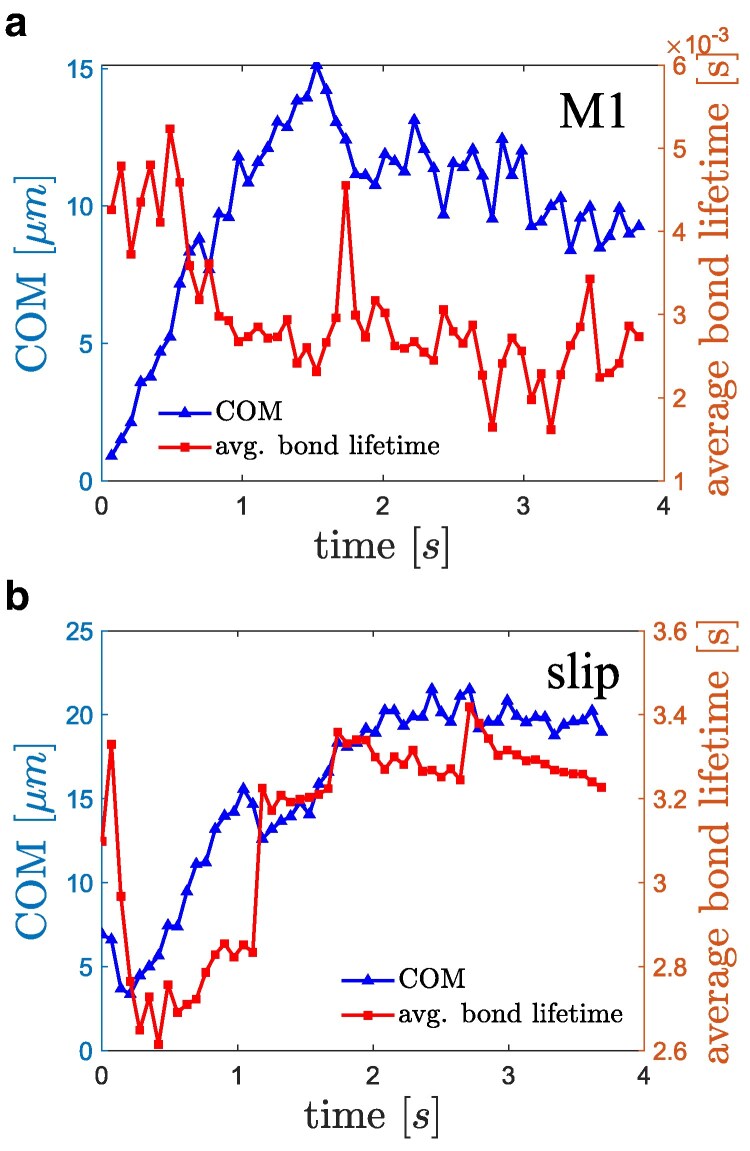
Average bond lifetime and the center-of-mass (COM) position of aggregate with respect to the wall as a function of time for a) catch-slip model M1 and b) slip model.

Differences in aggregate formation and dissociation described above are due to distinct bond lifetimes (or off-rates), which also depend on the length of bonds or stresses applied to them. Figure 7 shows the average lifetime of bonds within aggregates for the catch-slip model M1 and the slip model as a function of time. Note that the average lifetime of catch-slip bonds in Fig. 7(a) is about three orders of magnitude smaller than that for slip bonds in Fig. 7(b). For comparison, the average lifetime of catch-slip bonds is also about three orders of magnitude smaller than the dissociation time of several seconds for the whole aggregate [see Fig. 6(b)]. For reversible aggregation, it is intuitive that the average bond lifetime should be significantly smaller than the aggregate dissociation time, because many bonds need to be broken during dissociation, and new bonds can also be formed. Furthermore, as aggregates migrate away from the wall, the average lifetime of catch-slip bonds decreases, since flow stresses in the middle of the channel are smaller than at the wall. In contrast, the average lifetime of slip bonds increases as the aggregates migrate toward the channel center. Thus, the magnitude of bond lifetime and its dependence on applied stresses are crucial for the reversibility of VWF–platelet aggregation. Another important aspect for the reversibility of aggregation is the deactivation of VWF monomers. Within the near-wall region, even if a bond between a platelet and VWF monomer is broken, the VWF chain remains stretched and activated, so that a free binding site can quickly form a new bond. In contrast, within the dissociation zone, a freed monomer of VWF can get deactivated due to local chain collapse at low shear stresses. As a result, the dependencies of both bond lifetime and monomer activation/deactivation on local flow stresses determine whether VWF–platelet aggregates are reversible or not.

**Fig. 8.**
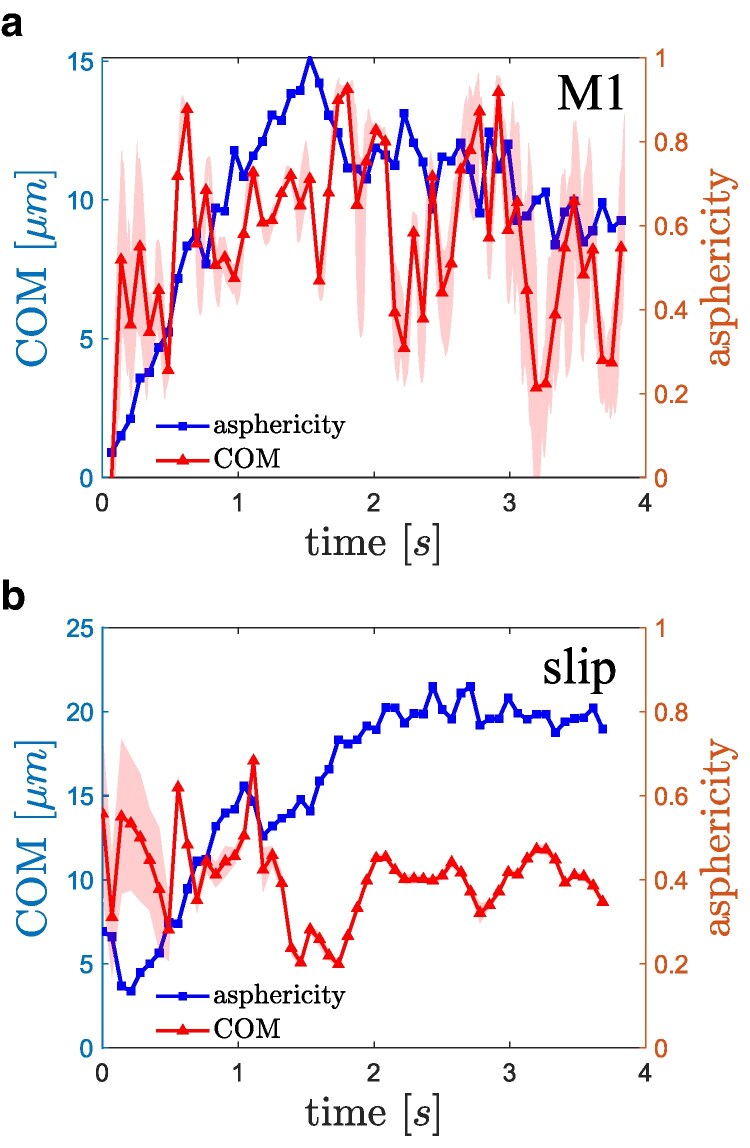
Asphericity and the position of aggregate center-of-mass (COM) position with respect to the wall as a function of time for a) catch-slip model M1 and b) slip model. The shaded areas represent local standard deviation.

Another interesting property is the deformability of formed VWF–platelet aggregates in flow. Figure 8 shows the asphericity of aggregates for the catch-slip model M1 and slip model as a function of time. Asphericity is computed as $\alpha =[(\lambda_{1}-\lambda_{2}{)}^{2}+(\lambda_{2}-\lambda_{3}{)}^{2}+(\lambda_{3}-\lambda_{1}{)}^{2}]/(2R_{g}^{4})$, where $\lambda_{1}$, $\lambda_{2}$, and $\lambda_{3}$ are the eigenvalues of the gyration tensor based on all particles within the aggregate and $R_{g}^{2}=\lambda_{1}+\lambda_{2}+\lambda_{3}$. The asphericity characterizes the deviation from spherical symmetry, such that it should be close to zero for a spherical aggregate, and to unity for a stretched configuration. For the catch-slip model M1 in Fig. 8(a), the asphericity first rapidly increases, as the aggregate grows, and then fluctuates around a value of α≃0.5−0.6, which indicates that the aggregate has a prolate shape and is rather soft, subject to significant stretching in flow. The fluctuations in *α* are due to local shear flow that periodically stretches and compresses the aggregate. For the slip model in Fig. 8(b), the asphericity is on average smaller than for the catch-slip model M1 and also has smaller fluctuations. This indicates that the aggregate for the slip model has a more spherical shape and is less deformable, which is expected due to persistent integrity of the irreversible aggregate.

**Fig. 9.**
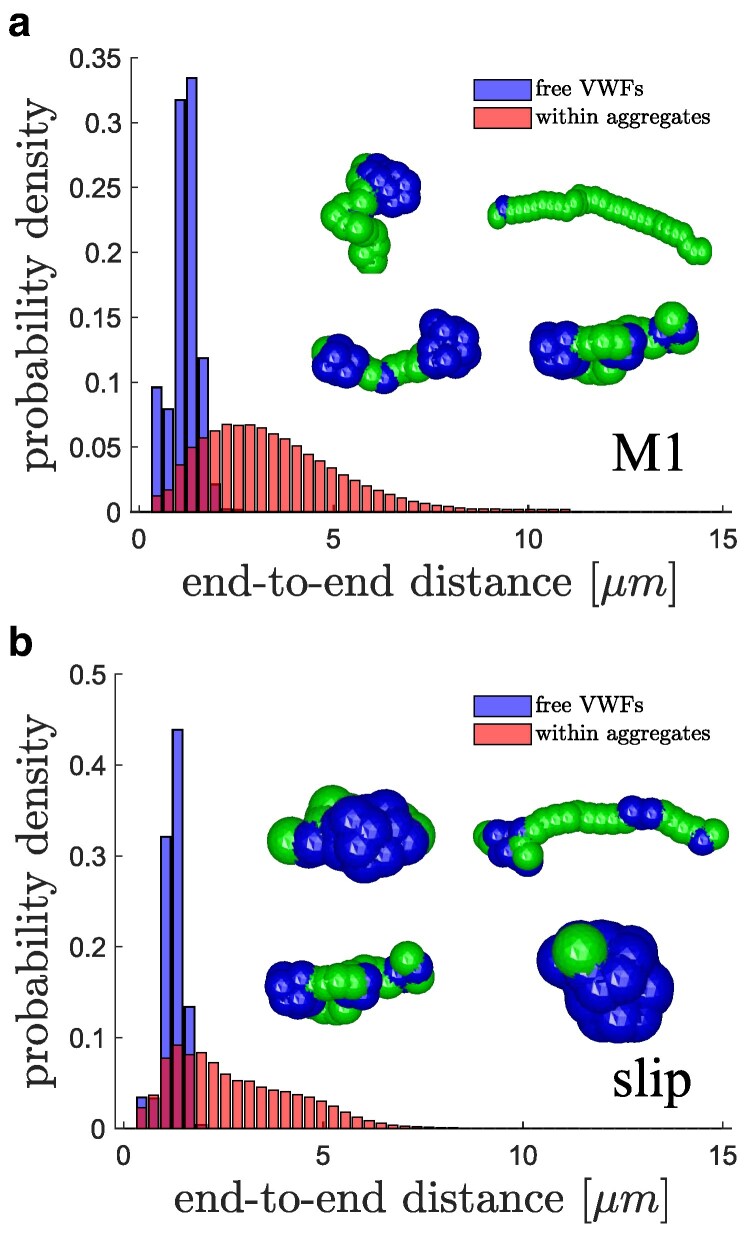
End-to-end distance distributions for both soluble VWFs and those within aggregates for a) catch-slip model M1 and b) slip model. Insets show a few exemplary configurations of VWF within aggregates, with globular, inactive parts (blue) and stretched, active parts (green).

Finally, Fig. 9 presents end-to-end distance distributions for both free VWFs and those within aggregates to characterize and compare VWF stretching under different conditions. Intuitively, the distributions of end-to-end distances for free VWFs (i.e. when they are not a part of any aggregate) are very similar for the catch-slip model M1 and the slip model, since blood flow conditions and the associated shear stresses are the same. However, VWFs within aggregates are much more stretched than freely flowing VWFs, as they frequently connect several platelets together. The typical VWF conformations in aggregates [see insets in Fig. 9] show moderate stretching and binding to only a few platelets. The distribution in Fig. 9(a) for VWFs within aggregates indicates that they are more stretched than those for the slip model in Fig. 9(b). This is due to the reversibility of aggregates in case of the catch-slip model M1, so that when the aggregates dissociate, they become more deformable, which results in additional stretching of VWF polymers.

## Discussion and conclusions

**Fig. 10.**
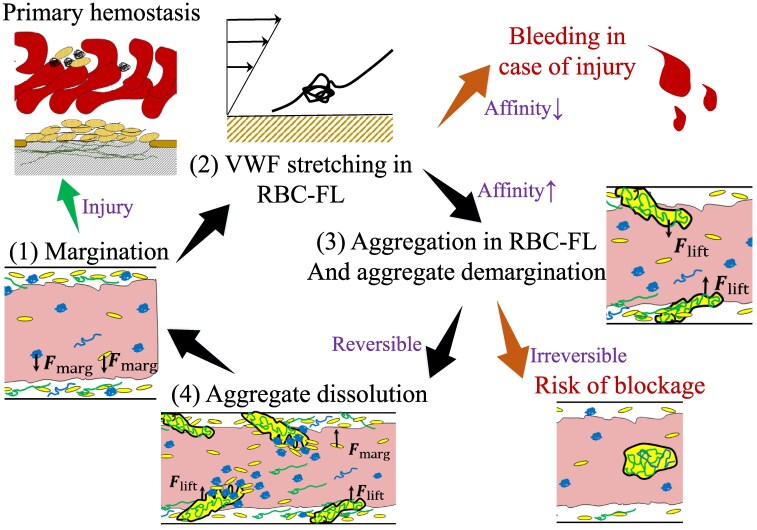
A comprehensive summary of platelet–VWF aggregation in blood flow. The aggregate formation process includes several stages, including (1) margination of platelets and VWF molecules, (2) VWF stretching in the RBC-FL, (3) aggregate growth within the RBC-FL, and (4) the migration away from the wall with a consecutive disaggregation under physiological conditions or possible retention of the aggregate in a pathological scenario. Note that this process is expected to be continuously repeated within the microvasculature.

We have studied the behavior of VWF–platelet aggregates in blood flow through microvessels. A comprehensive overview of the deduced mechanisms is shown in Fig. 10. To initiate aggregation, VWF molecules must be stretched by flow stresses and come into contact with flowing platelets. The stretching of VWF takes place primarily near the wall, where the shear stresses in Poiseuille flow are largest (35). Through the margination of VWFs and platelets into the RBC-FL, their concentration in the near wall layer might be substantially increased, which favors the formation of VWF–platelet aggregates as long as the activation of VWF can occur. As a result, VWF–platelet aggregates grow within the RBC-FL until they are large enough to be forced back into the bulk of blood flow. In the bulk of the Poiseuille flow, reversible aggregates may fall apart due reduced flow stresses, while in the case of irreversible aggregation, they may retain their content for a long time. This process is expected to continuously repeat with a periodic formation and dissociation of small VWF–platelet aggregates.

Under normal conditions, our model predicts the dissociation of aggregates when they are away from the walls, and therefore, are subject to reduced flow stresses. This is qualitatively consistent with the dissociation of wall-bound aggregates in the experiments (21, 23, 56), whenever the flow is significantly reduced or stopped. To corroborate the result that a reduction of flow stresses is the primary mechanism for disaggregation, we have also simulated blood under planar Couette flow, where the lower wall in our setup is kept stationary and the upper wall is moved with a prescribed velocity. The aggregation process for the Couette flow conditions initially resembles that for the pressure-driven Poiseuille flow. VWFs and platelets first marginate into the RBC-FL, where the formation of aggregates is observed. However, after the migration of large enough aggregates away from the wall, they show no disaggregation even for the case of catch-slip M1 bonds, since no reduction in flow stresses occurs in the bulk. Thus, the simulations confirm that a significant reduction in applied stresses is necessary for disaggregation.

There can be numerous deviations from the described process under some pathological conditions, with both reduced and enhanced aggregate formation. For instance, in vessels with low flow rates (e.g. venular flow), the shear stresses near vessel walls might be not sufficient for VWF stretching and activation, resulting in reduced formation of aggregates. Furthermore, when interactions between VWFs and platelets are too weak to sustain large enough aggregates under local flow conditions, the formation of aggregates can be significantly reduced. In this case, small floppy aggregates can still form, but they are generally very short lived. This can occur in certain types of von Willebrand disease (VWD) (14, 30) due to malfunctioning of VWF proteins. Moreover, reliable formation of VWF–platelet aggregates requires the presence of long enough VWF molecules, since short VWF chains do not stretch significantly at physiological shear rates and thus cannot result in proper aggregate formation.

On the other hand, the formation of VWF–platelet aggregates can also be significantly enhanced. For example, when VWF molecules are stretched and activated at lower shear stresses than those for normal VWF functioning, the formation of aggregates should also become possible at reduced flow rates. Furthermore, if the lifetime of bonds between VWFs and platelets is substantially increased, the dissociation of aggregates can also become significantly slower. This has been illustrated in our simulations by the catch-slip models M2 and M3, where the formed aggregates do not dissociate over the whole time of simulations. Theoretically, such VWF–platelet aggregates are still reversible, but the disaggregation is expected to occur over much longer time scales. These simulations can mimic a variety of pathological conditions under which the aggregation is significantly enhanced, leading to an increased risk of vessel blockages (10).

It is worthwhile to compare the main conclusions of our study with a previous simulation study (23) without RBCs, where platelets were modeled as spherical colloids. The presence of RBCs is essential for the margination of platelets and VWFs in the RBC-FL, which implies much higher wall-shear rates for a fixed flow rate, locally higher concentrations of VWFs and platelets near the wall, and thereby strongly enhanced aggregate formation. Furthermore, the presence of RBCs implies a blunt flow profile in the channel center, which favors aggregate dissociation. At low hematocrits, the margination of platelets and VWFs is significantly diminished, so that aggregate formation near a wall is expected to be strongly reduced. On the other hand, the size of aggregates near a wall (if they can form) may become a bit larger, since the thickness of the RBC-FL is larger at lower hematocrits. In summary, the presence of RBCs is essential and affects the hemostasis process both qualitatively and quantitatively.

It is also important to discuss possible limitations of the simulation model. Note that we study channel flow with concentrations of VWFs and platelets which are larger than under physiological conditions. The main reason for this choice is to accelerate the formation of VWF–platelet aggregates, making our simulations feasible. Since margination and stretching of individual VWFs should be nearly independent of their concentration, as this is not a cooperative process, the formation of VWF–platelet aggregates near the wall should be qualitatively similar for the case of reduced concentration of platelets and VWF, but slower due to a lower collision rate. Note that growing VWF–platelet aggregates remain stable at high shear rates near the wall, which is well supported by the simulations of linear Couette flow, suggesting that the aggregates can grow up to sizes required for leaving the RBC-FL. As a result, lower concentrations of VWFs and platelets are expected to slow down the formation of aggregates, but the described process of reversible aggregation should remain qualitatively unaffected. Furthermore, VWF–platelet aggregation can be significantly affected by various regulatory processes present in blood. For instance, VWF length is regulated by the protease ADAMTS13, which is able to cleave VWF molecules when the cleavage sites are accessible (normally when VWF is stretched) (14, 16). We did not consider possible cleavage of VWF, and the effect of reduced chain lengths, which are expected to reduce the tendency of aggregate formation. Clearly, the blood-flow geometry considered in simulations is relatively simple, in comparison with complex network-like structures in the microvasculature. Despite this simplification, our simulations qualitatively predict reversible aggregation in blood flow, though the aggregation process can be quantitatively different for a variety of geometries and flow conditions within the microvasculature. Finally, we did not consider the adhesion of aggregates at vessel walls, which would be expected at sites of injured endothelium. In such cases, we expect a stronger stretching of wall-bound VWFs, leading to efficient aggregate formation at the wall. Wall-bound aggregates should not spontaneously dissociate, as they are constantly subjected to large flow stresses near the wall.

Our simulations suggest the continuous formation and dissociation of small VWF–platelet aggregates within the microcirculation. This process has not been observed directly in experiments, though the formation of stationary wall-bound reversible aggregates was demonstrated in several experimental studies (21, 23, 56). It is clearly difficult to follow and observe unbound dynamic aggregates for a longer time, as they are quickly advected by the flow. It should be easier to observe already formed aggregates downstream, for which relatively long microchannels are required in in-vitro experiments to allow for aggregate formation. For the proof of concept, an elevated concentration of platelets and VWF would be advantageous to significantly accelerate the formation of VWF–platelet aggregates. Furthermore, microchannels employed in experiments should be small enough (presumably smaller than about 40--50μm in diameter) to avoid the obstruction of optical observation of aggregates by the presence of a large number of RBCs. In addition, flow rates in small channels are lower than in large channels, which can simplify reliable monitoring of VWF–platelet aggregates. In vivo observation of dynamic VWF–platelet aggregates is likely even more challenging than their monitoring in in vitro experiments.

We can only speculate whether the reversible formation and dissolution of such aggregates has some biological function. Undoubtedly, hemostasis is a finely tuned process that has to function for a wide range of local vascular geometries and flow conditions, since seemingly small changes in the hemostatic process can cause bleeding or undesirable thrombotic events. A possible function of reversible aggregation is the neutralization of aggregates which have spontaneously formed or detached from an existing stationary aggregate, thus preventing possible occlusion of microvessels—while at the same time recycling the components for further use. The formation of VWF–platelet aggregates may also affect the distribution of platelets and VWFs in the microvasculature, since the aggregates contain significant amounts of these two components. We hope that our simulation predictions will motivate further experimental studies in this area of research.

## Supplementary Material

pgaf375_Supplementary_Data

## Data Availability

The data underlying this article are available in the article, in its online supplementary material, and in the Zenodo repository at https://zenodo.org/records/17517292.
