# Supplementary material for: Reversible formation of von-Willebrand-factor–platelet aggregates in microvascular blood flow
Source: PNAS Nexus. 2025 Nov 29;4(12):pgaf375. doi: 10.1093/pnasnexus/pgaf375 (PMC12687593; doi:10.1093/pnasnexus/pgaf375)
Supplement: pgaf375_Supplementary_Data [file pgaf375_supplementary_data.zip › PNASNEXUS-PNASNEXUS-2025-00936R-s01.pdf]

Supplemental Material

Reversible formation of von-Willebrand-factor-platelet aggregates in microvascular blood flow

Alper Topuz,[1] Masoud Hoore,[1] Gerhard Gompper,[1] and Dmitry A. Fedosov[1,*]

[1] *Theoretical Physics of Living Matter, Institute for Advanced Simulation, Forschungszentrum Jülich, 52425 Jülich, Germany*

* d.fedosov@fz-juelich.de

I. DESCRIPTION OF MOVIES

Movie S1: Time-dependent formation of VWF-platelet aggregates in the RBC-FL. RBCs are drawn in red and platelets in yellow. VWFs are represented by blue (inactive) and green (active) monomers. For a better visibility, RBCs in the lower half of the channel are removed.

Movie S2: Dissociation of VWF-platelet aggregates for the catch-slip model M1, due to their migration away from the walls, with a significant reduction in flow stresses. RBCs are drawn in red and platelets in yellow. VWFs are represented by blue (inactive) and green (active) monomers. For a better visibility, RBCs in the lower half of the channel are removed.

Movie S3: Migration of irreversible VWF-platelet aggregates for the slip model toward the channel center, with no significant dissociation during the simulation time. RBCs are drawn in red and platelets in yellow. VWFs are represented by blue (inactive) and green (active) monomers. For a better visibility, RBCs in the lower half of the channel are removed.
